# The Reliability and Validity of Chinese Version of JSPE‐HP Among Doctors From Primary‐Level Clinics: A Cross‐Sectional Study

**DOI:** 10.1002/hsr2.71305

**Published:** 2025-11-17

**Authors:** Shumin Mai, Hongmei Wang, Lu Li

**Affiliations:** ^1^ The Institute of Social Medicine and Family Medicine, School of Medicine Zhejiang University Hangzhou China; ^2^ Zhejiang Shuren University Hangzhou China; ^3^ Zhejiang Urban Governance Research Center Hangzhou China

**Keywords:** confirmatory factor analysis, doctor behavior, empathy, reliability, validity

## Abstract

**Background and Aims:**

Primary‐level clinics play an important role in providing healthcare services and maintaining citizens' health in China. However, there is limited research on empathy among Chinese doctors in primary care settings. We will translate the Jefferson Scale of Physician Empathy for physicians and health professional (JSPE‐HP) into Chinese and evaluate its reliability and validity among doctors from primary‐level clinics and the condition of doctor empathy will also be evaluated.

**Methods:**

Two research assistants, fluent in both English and Chinese, were invited to evaluate our translation of the JSPE‐HP. From April to July in 2023, a cross‐sectional survey was then administered to a convenience sample of 459 doctors from primary‐level clinics in Zhejiang Province, China. The quality of the scale was tested using internal consistency reliability, composite reliability (CR), and convergent and discriminant validity.

**Results:**

The Chinese version of the JSPE‐HP scale comprises 20 items across three dimensions. The scale had an overall Cronbach's α of 0.85, and the CRs of the three dimensions were 0.88, 0.81 and 0.74 respectively, indicating satisfactory reliability. Average variance extracted ranged from 0.43 to 0.59 and the Chi‐squared difference between the constrained and free models was significant, indicating convergent and discriminant validity. The mean total score for the doctors was 110.52 (SD = 15.80). It was 75.43 after transformation.

**Conclusion:**

The reliability and validity of the scale is acceptable, and it could serve as an evaluation tool for doctors of Chinese background. The empathy level of doctors from primary‐level clinics should be improved further.

## Summary

- The Chinese Version of the Jefferson Scale of Physician Empathy (JSPE‐HP) exhibited acceptable reliability and validity, thus serving as a suitable evaluation tool for measuring doctor empathy from China.
- The empathy level of Chinese doctors from the primary‐level clinics should be further improved.

## 1 Background

The notion of empathy first emerged in the 19th century, with its philosophical and esthetic characteristics being delineated [1]. Since then it became popular in various fields, like psychology, management, education, and sociology. Empathy is a crucial element of interpersonal and social connection [1]. It emphasizes understanding, and care for others.

Empathy is one of the most important skills for medical staff. The book *CECIL Textbook of Medicine* mentions some common questions that patients care or ask when seeking medical care, like “How can I find a good doctor?,” “How can I find a good doctor whom I can afford?,” “How can I find a good doctor who cares about me as a person?,” and “How can I find a good doctor who will take the time to listen and understand?” [2]. These demonstrate that patients expect and long for care and understanding from their doctors. Meanwhile, doctors should possess the competency of empathy, which reflects patient‐center and human‐center [3, 4].

Relevant studies demonstrated that doctor empathy has positive influence on both themselves and patients. For example, doctor empathy can help to alleviate their occupational stress, reduce burnout, and improve working enthusiasm [5, 6]; it can also promote healthcare workers to understand patients' situation, enhance their sense of responsibility, and give more care and attention to their patients, which improves patients' trust and understanding [7]. In terms of the influence from doctor empathy on patients, doctor empathy can help to reduce patients' negative emotions, such as anxiety, worry, and depression, which can improve the mental health and self‐efficacy of patients during treatment [8, 9]. Regarding to patient treatment adherence, doctors deliver empathy, understanding, and care for their patients can increase patients’ trust, then encourage them to participate and cooperate during treatment, and increase their treatment adherence [10, 11].

Although the concept of empathy has been proposed in the early time, there is still no uniform definition of it. Different scholars proposed different concepts. The famous psychologist Rogers [12] defines empathy as “one's ability to accurately perceive the inner condition of another person as if one were the person being perceived, however without losing the assumption of ‘as if’.” In the medical field, Hojat et al. [1] hypothesized that, within the context of patient care, empathy is predominantly a cognitive attribute (i.e. cognitive empathy) as opposed to an emotional one. This hypothesis posits that empathy involves an understanding of the patient's experiences, concerns and perspectives, combined with a capacity to communicate this understanding.

Empathy is a concept that is defined in a variety of ways, thus resulting in a range of measurement tools. In the medical field, the series of empathy scales (e.g., the Jefferson Scale of Physician Empathy for Physicians and Health Professionals (JSPE‐HP) [13], the Jefferson Scale of Physician Empathy‐Student Version (JSPE‐S) [14], the Jefferson Scale of Physician Empathy‐Nursing Student Version (JEPE‐NS) [15], The Jefferson Scale of Patient's Perceptions of Physician Empathy (JSPPE) [16, 17]) developed by Hojat et al. from Jefferson University is widely used in many studies. Among them the Jefferson Scale of Physician Empathy for Physicians and Health Professionals (JSPE‐HP) [13], which is utilized to assess physician staff members' empathy popularly. And it was translated into difference kinds of language and widely used to evaluate medical staff's empathy in relevant studies [18, 19, 20]. The validation of JSPE‐HP, JEPE‐NS, and JSPE‐S has been primarily conducted among nurses [21], nursing students [22, 23], and medical students [24, 25]. However, studies among doctors, especially from primary‐level clinics among Chinese background are limited.

In China, the primary‐level clinics play an important role in the provision of healthcare services for the population, especially for those residing in rural, economically disadvantaged or remote mountainous regions. In recent years, China has undertaken a series of healthcare reform measures [26]. A large number of healthcare policies have been implemented with the aim of enhancing the quality of care provided by primary‐level clinics and ensuring that individuals have access to high‐quality medical services in their local area, thereby reducing the reliance on tertiary hospitals located in city centers. For instance, there has been a significant increase in the promotion of integrated healthcare systems, telemedicine, and family doctor services in recent years. Many chronic disease patients like those with hypertension, diabetes, stroke would like to go to primary‐level clinics to seek medical help because of its convenience. Therefore, doctors' behavior or performance, like showing kindness, understanding, care or patience, namely doctor empathy, may have important influence on patients' adherence or doctor‐patient relationship.

In general, this study will thus entail the translation of JSPE‐HP into Chinese and the subsequent evaluation of its reliability and validity among doctors working in primary‐level clinics. Meanwhile, the status and the general performance of doctor empathy will also be evaluated.

## 2 Methods

First, two research assistants, proficient in both English and Chinese, were invited to evaluate the translation of the JSPE‐HP. Second, a cross‐sectional survey was administered to a convenience sample of 459 doctors from primary‐level clinics in Zhejiang Province, China from April to July in 2023. The internal consistency reliability, composite reliability (CR), and convergent and discriminant validity were used to evaluate the quality of the scale.

### 2.1 Scale Translation

The Jefferson Scale of Physician Empathy for physicians and health professionals (JSPE‐HP) was developed by Hojat et al. [13] and has been widely used and validated in many studies related to doctor empathy. In 2009, Chinese researcher Ma and Li [21] developed Chinese version of JSPE‐HP and validated the quality of the scale among the clinical nurses.

In this study, we planned to invite doctors to participate in the investigation. After obtaining the authorization from Prof. Hojat and the Jefferson University (Supporting Information Table S2), two members (one PhD student and one professor) from the research team translated the scale into Chinese with comprehensive consideration of doctor's work characteristics and Chinese expression. Professor Ma was invited to undertake a comparative evaluation of the suitability and clarity of the translated items, focusing on the alignment between the Chinese and English expressions in terms of meaning and the congruence with the professional context of the doctors. Professor Ma also received an invitation to propose an alternative translation if deemed necessary. Then it was sent to a researcher who are familiar with both Chinese and English from the Jefferson University to help us to back‐translated the Chinese version into English version and assess concept equivalence. Items would be modified according to the suggestions provided by the evaluators. After a total of 3 rounds evaluation, the primary draft of the Chinese version of JSPE‐HP was established. The scale contains 20 items and three dimensions: perspective‐taking, compassionate care and standing in the patient's shoes. “Perspective taking” describes the physician's view of the patient's perspective, or the physician's view of the world from the patient's perspective. Compassionate care refers to a physician who understands their patients’ experiences and feelings. Standing in patient's shoes means the physician thinks like the patient. A 7‐point Likert scale was used to indicate agreement from 1 to 7 (reverse‐scored items were scored on: Strongly Disagree = 7 and Strongly Agree = 1. The other items were scored directly on their Likert weights. Strongly Disagree = 1; Strongly Agree = 7). The total score is the sum of all the item scores. The higher the score, the more empathic the behavioral orientation. Respondents must answer at least 16 (80%) of the 20 items; otherwise, their responses will be excluded from the data analysis. Missing values will be replaced with the mean score.

### 2.2 Scale Testing

#### 2.2.1 Participants

Doctors from primary‐level clinics in Zhejiang Province, China, were invited to participate in an online survey. The inclusion criteria were: (1) employees of the clinics; (2) holders of independent prescription rights; (3) proficient in the treatment of hypertension and/or diabetes; (4) willing to participate and sign the informed consent form.

#### 2.2.2 Investigation Tool

The questionnaire of this study consisted of two sections: the first comprised general information, while the second section incorporated a self‐evaluation of empathy. A range of demographic information was collected, including gender, age, marital status, and educational level. The measurement of self‐evaluation empathy was undertaken using the Chinese version of the JSPE‐HP, as translated in this study.

#### 2.2.3 Data Collection

In accordance with the sample calculation formula: $n={\left(\frac{u_{\alpha ⁄2}\sigma }{\delta }\right)}^{2}$ [27] and in consideration of the findings of Ma and Li [21] which demonstrated that the empathy score of 300 clinical nurses was 105.94 (SD = 12.44) when the quality of the JSPE‐HP in China was assessed. After putting $u_{\alpha ⁄2}$ = 1.96,σ = 12.44,δ (tolerance) = 0.1 × 12.44 into the formula and considering 10% questionnaire loss rate, at least 427 questionnaires need to be collected. According to 2022 Statistical Yearbook of Zhejiang Province in China, the cities in Zhejiang Province are divided into three groups based on the GDP level. The GDP of high‐level, medium‐level, and low‐level groups were exceeded 1 trillion yuan (included two cities), between 300 and 700 trillion yuan (included six cities) and between 300 and 700 trillion yuan (included three cities), respectively. Hangzhou and Ningbo cities, Taizhou and Jinhua cities, Quzhou and Lishui cities, from high, medium and low level GDP groups respectively, Two cities from each group would be selected according to convenience and accessibility (eg. primary‐level clinics has willingness to cooperate). We planned to invite doctors from 10 primary‐level clinics of each selected city to participate in the survey. In China, many doctors in primary‐level communities would provide medical services in the villages or remote areas, which are far away from the city or urban centers. To invite more doctors to participate in the investigation, we mainly collect the data through the online questionnaire. We designed the online questionnaire on “Yibiaoda” website which is one of the most popular and safest online questionnaire design platform in China. Participants could choose the answer of the questions, sign their name for the agreement to participate in the survey. A coordinator from each research site would help to deliver the online questionnaire link to their working group through the popular communication media like Wechat, QQ, or Dingding. From April to July in 2023, we spent 1 to 2 weeks to collect the online questionnaire of each research site (the independent links for each research site were designed). After finishing the questionnaires collection, the online questionnaire links would be closed by the research member (SMM) then no other participants could fill in the questionnaire.

#### 2.2.4 Data Management

A total of 504 questionnaires were distributed, and 459 were returned, yielding a response rate of 91.07% (459/504). Questionnaires from 45 participants was excluded on the basis of two criteria. Firstly, more thn 4 items of the total scale are not answered, it will be considered invalid fewer items (response rate is less than 80% of the total 20 items). Secondly, participants who had not responded to the questionnaire in a serious manner were also excluded. This latter criterion was applied in instances where the participant had scored outside the normal variation or had submitted a majority of strongly agree or strongly disagree responses.

#### 2.2.5 Statistical Analysis

The reliability and validity of the Chinese version of JSPE‐HP were evaluated. To assess the internal consistency reliability, Cronbach's α values for the total scale were calculated by SPSS software version 20.0. It was considered that a Cronbach's α coefficient greater than 0.70 would be satisfactory [28, 29, 30]. As JSPE‐HP possesses its own unique structure and dimensions, we conduct a confirmatory factor analysis (CFA) to examine and validate its structural characteristics by AMOS 21.0. The quality of CFA model was evaluated by several different indices, including the goodness‐of‐fit index (GFI), the adjusted GFI (AGFI), the normed fit index (NFI), the comparative fit index (CFI), the parsimony goodness‐of‐fit index (PGFI), and the root mean square error of approximation (RMSEA). The CFA model is deemed acceptable when the GFI, AGFI, NFI, and CFI are greater than 0.90, the PGFI is greater than 0.50 and the REMEA is less than 0.08 [30, 31]. Meanwhile, CFA conducted by AMOS can also be used to evaluate the composite reliability (CR) and convergent and discriminant validity of the scale [28, 30]. Composite reliability(CR) is utilized to assess the consistency of latent construct indicators, thereby demonstrating the extent to which measurement indicators are shared across latent variables. It was considered that a CR greater than 0.70 would be satisfactory [28, 30]. The average variance extracted (AVE) is indicative of the extent to which latent variables can explain the measurement indicators. The present study employed AVE to ascertain convergent validity. It has been demonstrated that, upon attaining a value of 0.50 or higher, the indicator variable is capable of reflecting its latent variable with a high degree of effectiveness [30, 32, 33]. To assess discriminant validity, a series of 2‐factor confirmatory models were employed, incorporating χ2 difference tests for each pair of constructs. In the context of the present study, the constrained model, which imposed a restriction on the phi coefficient to equal 1, was compared with a free model devoid of this constraint. In all cases, a significant χ² difference was indicated, thereby demonstrating that discriminant validity has been proven [28, 30].

The CFA analysis was conducted by utilizing AMOS 21.0 software, whereas the other analysis was conducted by means of SPSS software version 20.0. Meanwhile, the scale's total, three dimensions and each item score (mean and standard deviation) were utilized to illustrate the general status of primary health care doctors in terms of empathy. Meanwhile, the raw score for doctor empathy was derived by summing the item scores and converting them to values between 0 and 100 [34]. It was then recalculated across the dimension as follows: Transformed score = ([actual raw score – lowest possible raw score]/possible raw score range) × 100. And two‐sided *p* < 0.05 was considered significant in all analyzes.

## 3 Results

### 3.1 Participant Characteristics

The investigation encompassed a total of 46 primary‐level clinics, including 21 community health centers and 25 township health centers. There were 196 doctors from urban/village area and 263 from the city area. There were 258 (56.21%,258/459) female participants. The percentage of the participants who had received master degree or above was 81.70% (375/459). The average age and working years of the participants were 40.87 and 16.88 years, respectively (Supporting Information Table S1).

### 3.2 Reliability and Validity of the Scale

The internal consistency of the empathy scale was examined by calculating Cronbach's coefficient alpha. The reliability coefficient for the Chinese version of Jefferson Scale of Physician Empathy for Physicians and Health Professionals (JSPE‐HP) was determined to be 0.85, indicating an internal consistency within the scale.

**Figure 1:**
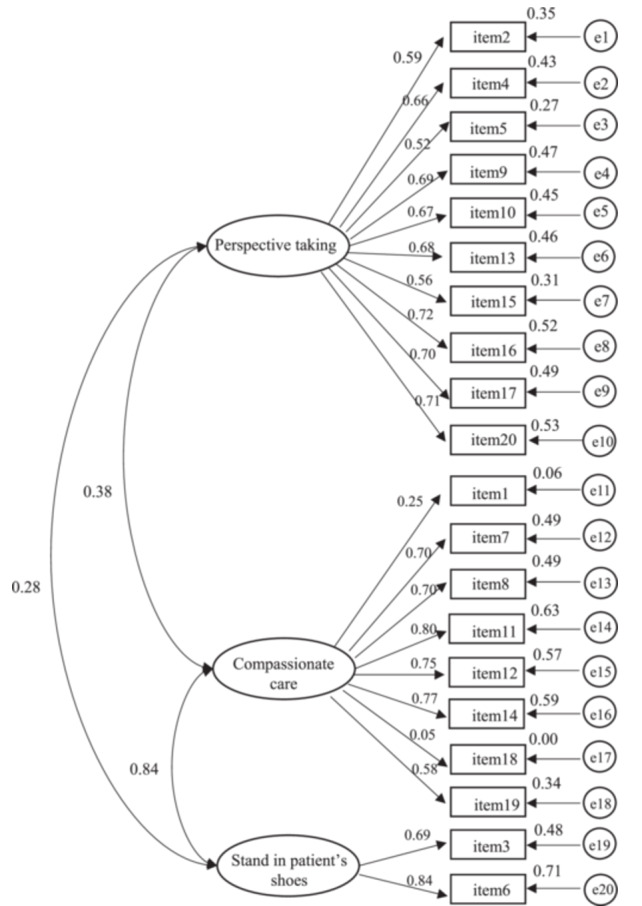
CFA model of Chinese version of JSPE‐HP. (1) CFA, confirmatory factor analysis; (2) e, error; (3) in the model the latent variables were: perspective taking, compassionate care, stand in patient's shoes; observed variables were: doctor self‐evaluation data (7‐point Likert scale) of items 1 to 20; (4) values between 3 latent variables (perspective taking, compassionate care and stand in patient's shoes) are correlations; values between 3 latent variables and their observed variables are standardized regression weights (e.g., value 0.59 between perspective taking and item2, value 0.25 between compassionate care, value 0.69 between stand in patient's shoe and item 3); (5) the other values (such as 0.35, 0.43, 0.27) are squared multiple correlations of 20 observed variables, they were used to reflect the error degree of the observed variables; when it is small, the error is large.

The CFA model was established on the theoretical structure of the scale. The analysis yielded the following results: χ2 = 491.37, df = 224, χ2/df = 2.94, GFI = 0.90, AGFI = 0.87, and RMSEA = 0.07 with a 90% CI of 0.06 to 0.07, NFI = 0.87, CF = 0.91, PGFI = 0.71. The majority of indexes were found to be in accordance with or in proximity to the established criteria, thereby indicating the model's acceptability. The fit indices indicated an adequate fit of the model to the data (Figure 1).

With the exception of items 1 and 18, the regression weights of the remaining items exceeded 0.40. The values of the CRs of the perspective‐taking dimension, the compassionate care dimension, and the stand in patient's shoes dimension were 0.88, 0.81, and 0.74, respectively. These values are all in excess of 0.70. The AVEs of the aforementioned latent variables were 0.43, 0.40, and 0.59, respectively. The dimension of the stand in patient's shoes demonstrated a value in excess of 0.50, while the remaining two dimensions reported values less than 0.50. To assess discriminant validity, a χ2 difference test was performed on the constrained model, which constrained the phi coefficient to equal 1, with a free model without this constraint. In all cases, the χ2 difference was found to be significant, indicating satisfactory discriminant validity (see Tables 1 and 2).

**Table 1:** CRs, and AVEs of the scale.

Items	Standard loading	S.E.	CR	AVE
Perspective taking			0.88	0.43
Item2	0.59	0.65		
Item4	0.66	0.57		
Item5	0.52	0.73		
Item9	0.69	0.53		
Item10	0.67	0.55		
Item13	0.68	0.54		
Item15	0.56	0.69		
Item16	0.72	0.48		
Item17	0.70	0.51		
Item20	0.73	0.47		
Compassionate care			0.81	0.40
Item1	0.25	0.94		
Item7	0.70	0.51		
Item8	0.70	0.51		
Item11	0.80	0.37		
Item12	0.75	0.43		
Item14	0.77	0.41		
Item18	0.05	1.00		
Item19	0.58	0.66		
Stand in patient's shoes			0.74	0.59
Item3	0.69	0.53		
Item6	0.84	0.29		

Abbreviations: AVE, average variance extracted; S.E, standard errors.

**Table 2:** Chi‐square difference test between constrained models and free models.

Each pair of the constructs	Free model			Constrained model			Chi‐square difference of the two models	df difference of the two models	*p*
	χ^2^	df	*P*	χ^2^	df	*P*			
PT and CC	457.88	134	< 0.001	643.64	135	< 0.001	185.76	1	< 0.001
PT and PS	243.773	53	< 0.001	343.485	54	< 0.001	99.712	1	< 0.001
CC and PS	82.115	34	< 0.001	86.872	35	< 0.001	4.76	1	0.029

Abbreviations: CC, compassionate care; PS, stand in patient's shoes dimension; PT, perspective taking dimension.

### 3.3 Scores of Doctor's Empathy

The mean total score for the 459 doctors was 110.52 (SD = 15.80). The mean scores for the other three dimensions were 62.88 (SD = 7.06), 37.79 (SD = 9.94) and 9.85 (SD = 3.57) respectively. After transformation (convert the raw score to values between 0 and 100), the total score of doctor empathy was 75.43. In terms of the three dimensions, perspective taking got the highest score followed by the dimension of stand in patient's shoes, and compassionate care (Table 3).

**Table 3:** The total score and the dimension scores of the doctor's empathy.

	Skewness	Kurtosis	Mean (raw score)	SD	Transformed score
Total score	−0.52	−0.73	110.52	15.80	75.43
Perspective taking	−1.03	0.64	62.88	7.06	88.13
Compassionate care	−1.05	0.95	37.79	9.94	62.06
Stand in patient's shoes	−0.54	−0.71	9.85	3.57	65.42

In terms of each items' score of the scale. Except for item 1 (“understanding of how my patients and their families feel does not influence medical or surgical treatment”) and 18 (“do not allow to be influenced by strong personal bonds between patients and their family members”), the mean scores of other items were greater than 4. Both item 1 and item 18 belong to the compassionate care dimension. The items that got the scores that rank top three were item 4 (“understanding patients' body language as important as verbal communication in caregiver‐patient relationships”), item 2 (“my patients feel better when I understand their feelings”), and item 9 (“try to imagine myself in my patients’ shoes when providing care to them”). These three items belong to perspective taking dimension. However, compared with other items, item 3, item 1 and item 18 got the lower scores. Item 3 is included into stand in patient's shoes dimension (Table 4).

**Table 4:** Mean scores of each items in the scale.

Items	Mean	SD	Rank
(−)1	3.04	2.20	19
2	6.57	0.78	2
(−)3	4.91	2.01	18
4	6.64	0.74	1
5	5.84	1.27	11
(−)6	4.95	2.00	17
(−)7	5.11	2.03	16
(−)8	5.21	2.05	15
9	6.48	0.87	3
10	6.36	0.92	6
(−)11	5.44	1.91	12
(−)12	5.40	2.06	13
13	6.04	1.17	9
(−)14	6.08	1.67	8
15	5.92	1.51	10
16	6.22	1.07	7
17	6.44	0.96	4
(−)18	2.30	1.47	20
(−)19	5.22	1.88	14
20	6.37	0.93	5

*Note:* “(−)” are reverse scored items (i.e., strongly agree = 1, strongly disagree = 7), while the other items are directly scored on their Likert weights (i.e., strongly disagree = 1…strongly agree = 7); in accordance with the scale authorization requirements established by the Jefferson University, the presentation of content is limited to a maximum of five items. Consequently, the content of items 1 and 18, items 2, 4, and 9 is primarily displayed. It is noteworthy that items 1 and 18 received low scores, while items 2, 4, and 9 received high scores: item 1: “understanding of how my patients and their families feel does not influence medical or surgical treatment”; item 18: “do not allow to be influenced by strong personal bonds between patients and their family members”; item2: “my patients feel better when I understand their feelings,” item 4: “understanding patients’ body language as important as verbal communication in caregiver‐patient relationships”; item 9: “try to imagine myself in my patients’ shoes when providing care to them.”

## 4 Discussion

### 4.1 The Chinese Version of the JSPE‐HP is of Good Quality and can be Used to Evaluate Empathy Among Doctors From Primary‐Level Clinics

The reliability (internal consistency reliability and CR) and validity (convergent and discriminant validity) of the scale were tested. The internal consistency reliability was tested by calculating Cronbach's α values. The findings demonstrated that Cronbach's α of the Chinese version of JSPE‐HP was 0.85, greater than the criteria of 0.70 [28, 29, 30]. The CRs of the three dimensions: perspective‐taking, compassionate care and stand in patient's shoes, were 0.88, 0.81, and 0.74, respectively. These values met the 0.70 criterion [28, 30], indicating good CR. In all cases, the chi‐squared difference was found to be having statistical significance, thus indicating that the scale possesses acceptable discriminant validity [28, 30]. The AVEs of the three dimensions were 0.43, 0.40, and 0.59, respectively. The AVE of the third dimension, stand in patient's shoes, was greater than 0.50, while the other two dimensions, perspective taking dimension and compassionate care, were little lower than 0.50. The regression weights of the two items, namely item 1 and item 18, were lower than 0.40. However, the analysis result demonstrated that the regression weights of the other items were above 0.40, and the scale had satisfied Cronbach's α and CRs. Thus, we decided to retain all the items.

Hojat et al. [1] evaluated the reliability and validity of JSPE‐HP among 704 physicians. The result demonstrated that the total score was 120 (SD = 11.9), and Cronbach's alpha was 0.8. Ma and Li [21] evaluated the quality of the JSPE‐HP among 300 clinical nurses. The resultant mean score for the nurses was 105.94 (SD = 12.44), with Cronbach's alpha being 0.80. This study demonstrated that the mean score of the 459 doctors was 110.52 (SD = 15.80). The score was lower than those observed by Hojat, but higher than those reported by Ma and Li.

### 4.2 The Perspective‐Taking Dimension Gains the High Score

With regard to the scores obtained in the three dimensions, the highest score was achieved in the dimension of perspective‐taking, then followed by the dimension of standing in patient's shoes and compassionate care.

In accordance with the empathy connotation proposed by Hojat et al. [1] in the context of patient care, empathy is a predominantly cognitive attribute. In contradistinction to emotional empathy, it emphasizes the comprehension of patients' experiences, concerns and perspectives. While emotional empathy mainly refers to physicians' emotional response to patients' pain and suffering, such as feeling happy or sad, similar with what patients' feeling. During disease treatment, healthcare professionals must maintain calm and objective. They should recognize and understand the patient's pain and suffering, but avoid being influenced by patients' strong emotions [1]. In this study, perspective taking dimension gain the highest score, which indicates that many doctors can maintain calm and objective when facing patients' emotion or feeling expression.

### 4.3 Doctors From Primary‐Level Clinics Still Need to Improve Their Empathy Levels

The analysis results showed that in terms of the total score, empathy of doctors from primary‐level clinics still needed to be improved. The high empathy ability will be beneficial to patient disease treatment as well as doctors themselves.

It has been demonstrated by several studies that there is a discrepancy between the patient's perception and the physician's self‐assessment. For instance, a study of doctors' service attitudes showed that 83.27% (234/281) of them self‐assessed that they would provide their patients with clear explanations until they fully understand. However, only 63.49%(153/241) of patients believed that doctors would conduct this behavior [35]. Moreover, 64.73% (156/241) of patients reported that they had received encouragement from their doctors, while 82.21% (231/281)of doctors reported that they would encourage their patients [35]. It has been demonstrated that when doctor demonstrate active empathy, including doctors showing concern for their patients, providing clear explanations, offering appropriate comfort and reassurance [9, 10, 36], patients are more likely to perceive this, thereby narrowing the gap between patient perception and self‐evaluation on the part of the doctor. Thus, patients will be more likely to adhere to medical advice and suggestions when they feel cared for and understood by their doctors, which will have a positive influence on patients' health and the effectiveness of their treatment. Furthermore, in China many older patients such as those with chronic diseases would visit primary‐level clinics because of its convenient and low prices. Older adults, who usually suffer from cognitive ability decline with ages, having more difficulty in comprehending medical knowledge compared with young people. Doctors need to explain the treatment methods to them more patiently and clearly, which will benefit to their chronic diseases treatment adherence. Meanwhile, doctor empathy will also bring positive influence on themselves, such as relieving their occupational stress [5, 6], and facilitating their sense of working responsibility and accomplishment [7, 37]. Our study result demonstrated that the empathy level of doctors from primary‐level clinics still needed to be improved. Empathy is one of the most important communication skills of doctors. Relevant studies indicate that empathy education or training can help to increase doctor empathy skill, measures like standardized patients, non‐verbal language communication with patients (such as touch or pat) [38], narrative medicine [39]. Currently, compared with professional knowledge and skills, the content of medical humanities and professional ethics is often being ignored in medical education or vocational training programs [40]. Empathy has positive influence on both doctors and patients and plays an important role during treatment process. Therefore, doctors should not only focus on increasing their technical skills but also pay more attention to their humanistic care ability.

### 4.4 Limitation

Despite the translation of the JSPE‐HP into Chinese and the subsequent evaluation of its reliability and validity, the participants were selected from primary‐level clinics. Consequently, it is not possible to generalize the results to the entire population of doctors in China. Further studies should be conducted in multiple regions and in different types of hospitals, and more doctors should be invited to evaluate their empathy fully.

## 5 Conclusion

This study translated JSPE‐HP into Chinese and validated it among doctors from primary‐level clinics. The analysis result indicated that the scale demonstrated acceptable reliability and validity, thereby suggesting its potential as a tool for evaluating doctor empathy within the context of Chinese primary healthcare.

## Author Contributions

**Shumin Mai:** conceptualization, investigation, writing – original draft, methodology. **Hongmei Wang:** writing – review and editing. **Lu Li:** conceptualization, writing – review and editing.

## Ethics Statement

This study was approved by the Ethical Review Committee of Zhejiang University School of Public Health (ZGL202209‐9). Informed consent was signed by all participants at the time of investigation. All methods were performed in accordance with the relevant guidelines and regulations.

## Conflicts of Interest

The authors declare no conflicts of interest.

## Transparency Statement

The lead author Lu Li affirms that this manuscript is an honest, accurate, and transparent account of the study being reported; that no important aspects of the study have been omitted; and that any discrepancies from the study as planned (and, if relevant, registered) have been explained.

## Supporting information


**Supporting Information Table S1:** Participants' Characteristics (*n* = 459). **Supporting Information Table S2: Authorisation of JSPE‐HP**.

## Data Availability

The data that support the findings of this study are available from the corresponding author upon reasonable request. The data sets generated during and/or analyzed during the current study are available from the corresponding author on reasonable request. After getting the authorization from the Thomas Jefferson University, the JSPE‐HP, both the original English version and the Chinese version, can be used.
