# Supplementary material for: The Reliability and Validity of Chinese Version of JSPE‐HP Among Doctors From Primary‐Level Clinics: A Cross‐Sectional Study
Source: Health Sci Rep. 2025 Nov 17;8(11):e71305. doi: 10.1002/hsr2.71305 (PMC12623455; doi:10.1002/hsr2.71305)
Supplement: Supplementary file 1 — Supporting Information Table S1: Participants' Characteristics (n = 459). Supporting Information Table S2: Authorisation of JSPE‐HP. [file HSR2-8-e71305-s001.docx]

**Supplementary**

**STable 1** Participants’ Characteristics (n=459）

|  |  | Frequencies (n) | Percentage (%)/ Mean±SD |
| --- | --- | --- | --- |
| Area | Urban/village | 196 | 42.70 |
|  | City | 263 | 57.30 |
| Gender | Male | 201 | 43.79 |
|  | Female | 258 | 56.21 |
| Age |  | 459 | 40.87±8.61 |
| Marital status | Single | 63 | 13.73 |
|  | Married | 396 | 86.27 |
| Educational level | Below Master | 84 | 18.30 |
|  | Master and above | 375 | 81.70 |
| Salary | <5000 RMB | 181 | 39.43 |
|  | ≥5000 RMB | 278 | 60.57 |
| Working year |  | 459 | 16.88±9.53 |


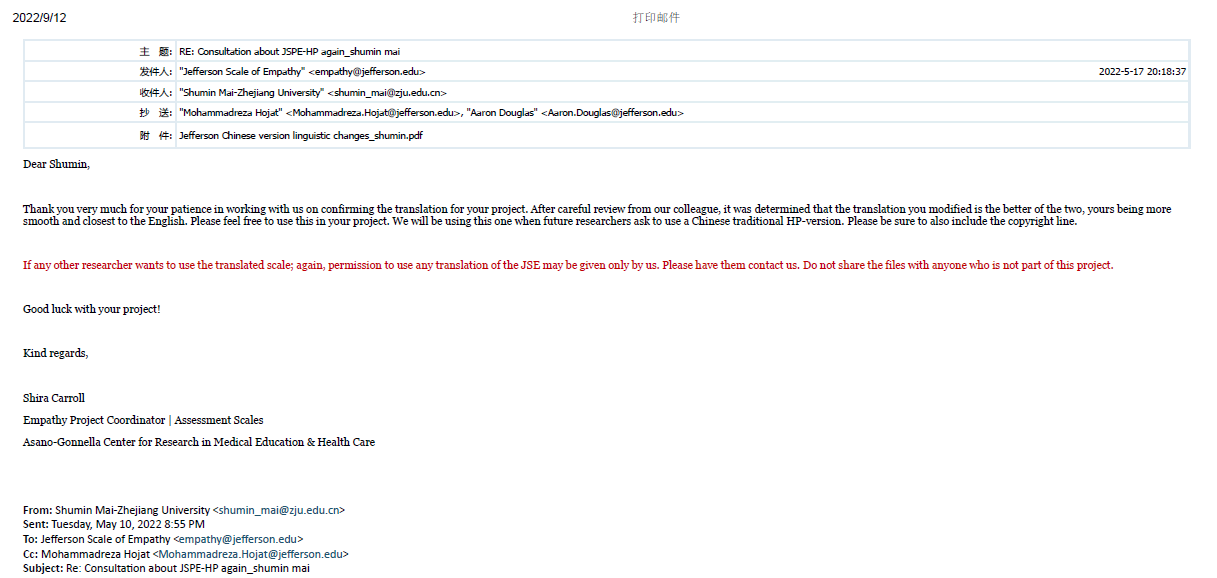
**S2 Authorisation of JSPE-HP**
